# RFLMDA: A Novel Reinforcement Learning-Based Computational Model for Human MicroRNA-Disease Association Prediction

**DOI:** 10.3390/biom11121835

**Published:** 2021-12-05

**Authors:** Linqian Cui, You Lu, Jiacheng Sun, Qiming Fu, Xiao Xu, Hongjie Wu, Jianping Chen

**Affiliations:** 1 School of Electronic and Information Engineering, Suzhou University of Science and Technology, Suzhou 215009, China; cui_youzhangjing@163.com (L.C.); sunjiacheng33@163.com (J.S.); xuxiao@post.usts.edu.cn (X.X.); hongjiewu@usts.edu.cn (H.W.); 2 Jiangsu Province Key Laboratory of Intelligent Building Energy Efficiency, Suzhou University of Science and Technology, Suzhou 215009, China; alanjpchen@yahoo.com; 3 Suzhou Key Laboratory of Mobile Network Technology and Application, Suzhou University of Science and Technology, Suzhou 215009, China; 4 School of Architecture and Urban Planning, Suzhou University of Science and Technology, Suzhou 215009, China; 5 Chongqing Industrial Big Data Innovation Center Co., Ltd., Chongqing 400707, China

**Keywords:** laplacian regularized least squares, neighborhood regularized logistic matrix factorization, Q-learning, collaborative matrix factorization, human microRNA-disease association

## Abstract

Numerous studies have confirmed that microRNAs play a crucial role in the research of complex human diseases. Identifying the relationship between miRNAs and diseases is important for improving the treatment of complex diseases. However, traditional biological experiments are not without restrictions. It is an urgent necessity for computational simulation to predict unknown miRNA-disease associations. In this work, we combine Q-learning algorithm of reinforcement learning to propose a RFLMDA model, three submodels CMF, NRLMF, and LapRLS are fused via Q-learning algorithm to obtain the optimal weight S. The performance of RFLMDA was evaluated through five-fold cross-validation and local validation. As a result, the optimal weight is obtained as S (0.1735, 0.2913, 0.5352), and the AUC is 0.9416. By comparing the experiments with other methods, it is proved that RFLMDA model has better performance. For better validate the predictive performance of RFLMDA, we use eight diseases for local verification and carry out case study on three common human diseases. Consequently, all the top 50 miRNAs related to Colorectal Neoplasms and Breast Neoplasms have been confirmed. Among the top 50 miRNAs related to Colon Neoplasms, Gastric Neoplasms, Pancreatic Neoplasms, Kidney Neoplasms, Esophageal Neoplasms, and Lymphoma, we confirm 47, 41, 49, 46, 46 and 48 miRNAs respectively.

## 1. Introduction

MicroRNA (miRNA) is a type of single-stranded endogenous non-coding RNA. It is composed of approximately 20~25 nucleotides [1] and mainly acts as a key regulator of genes expressed at the post-transcriptional level. It mainly exerts its biological functions by influencing the expression of target genes, if miRNA induces messenger RNA degradation, translation inhibition, or other morphological regulation mechanisms, the expression of target genes to be inhibited. Researchers have found that miRNAs exist in various eukaryotes and prokaryotes, they are involved in regulating many life processes of organisms, including a series of biological life processes, for example cell growth and development and the formation of vital organs. The abnormal modulation of miRNAs can lead to the development of numerous complex human diseases [2,3,4,5,6], such as cancer. Therefore, Studying the relationship between miRNAs and diseases is crucial to improve the treatment of complex diseases.

miRNA as a pathogenic factor for many complex diseases has the ability to accurately and efficiently identify miRNA-disease associations, which will help people to understand the pathogenesis of complex diseases and provide useful help for disease prevention and treatment. In the early stages, researchers mostly carried out miRNA-disease association prediction by biological experiments. But traditional biological experiments have some drawbacks such as the small scale, the large investment in manpower and material resources, the long experiment period, and the existence of limitations [7]. Due to the rapid advances in biotechnology, massive data has been generated in the field of biology. The computational method of bioinformatics come into being [8]. It not only points out the direction of traditional experiments to a certain extent, but also further reduces the cost of traditional biological experiments.

So far, predecessors have proposed many methods [9,10,11,12] to forecast miRNA-disease associations. In 2010, Jiang et al. [13] proposed a method. They fused data from multiple sources through a naive Bayesian model, via disease-gene association and miRNA-target gene association, they predicted the similarity score between the disease and each miRNA. The highest scoring miRNAs were those associated with the disease. Chen et al. [14] proposed a model in 2012, which is RWRMDA. However, their consideration is insufficient, the prediction performance is poor. In 2013, Xuan et al. [15] proposed HDMP, which is a computational model via weighted K nearest neighbors, but it did not predict unknown diseases which are involved with miRNAs. In this year, Shi et al. [16] used disease-gene associations and miRNA-target gene associations to perform random walks on the protein-protein interaction (PPI) network. In this way, they can get predicted results. Subsequently, Chen et al. [17] proposed a predictive method called RLSMDA in 2014, it is a novel approach and a semi-supervised globalization model, yet it did not consider the topological information of the miRNA-disease association network. In 2016, Liu et al. [18] built a more complete heterogeneous network by fusing multiple data sources, in predicting the correlation between miRNAs and diseases, they used a random walk algorithm. Via the same data, Chen et al. [19,20] successively proposed two methods to predict miRNA-disease associations in 2016. First, they proposed WBSMDA model, which calculates the Gaussian similarity score between miRNA and disease and uses it as miRNA-disease association prediction scores. Later, The HGIMDA method is proposed. It constructs a heterogeneous network and iterative updates are performed using optimization functions. In this way, they predicted the unknown connection between miRNAs and diseases. Comparing these two methods, the latter has faster and more effective characteristics. Based on a biological hypothesis that the functional similarity of miRNAs is positively correlated with similar phenotypes of diseases, a distribution model for hypergeometric computation is proposed in 2017. Jiang et al. [21] construct a miRNA functional similarity network and a known miRNA-disease association network, disease phenotypic similarity was used to express disease similarity, and disease-associated miRNAs were predicted by a hypergeometric distribution scoring system. But there is a limit to the amount of information that can be used to build a network. In 2018, Jiang et al. [22] proposed the FKL-Spa-LapRLS model, it learns through the Fast Kernel Learning (FKL) model, which is a combination of miRNA similarity kernels and disease similarity kernels, next then noise is removed by sparse kernels (Spa), finally LapRLS is used to find miRNA-disease associations. In 2020, Ding et al. [23] proposed a new model to predict miRNA-disease association through a hypergraph regularized bipartite local model (HGBLM) based on a hypergraph embedded Laplacian support vector machine (LapSVM).

In this paper, we combined Q-learning algorithm of reinforcement learning to propose RFLMDA model. The three sub-models were used, namely CMF [24], NRLMF [25], and LapRLS [26], which were fused via Q-learning algorithm to obtain the optimal weight S. The performance of RFLMDA was evaluated through five-fold cross-validation and local validation. As a result, the optimal weight was obtained as S (0.1735, 0.2913, 0.5352), and the AUC was 0.9416. By comparing the experiments with other methods, it is proved that RFLMDA model has better performance.

In order to further validate the predictive performance of RFLMDA, we use eight diseases for local verification and perform case study on three common human diseases. Consequently, all the top 50 miRNAs associated with Breast Neoplasms and Colorectal Neoplasms have been confirmed. Among the top 50 miRNAs related to Pancreatic Neoplasms, Colon Neoplasms, Gastric Neoplasms, Kidney Neoplasms, Esophageal Neoplasms and Lymphoma, we confirm 49, 47, 41, 46, 46, and 48 miRNAs respectively.

## 2. Materials and Methods

### 2.1. Human miRNA-Disease Associations

This paper downloads the required data from HMDD v2.0 (http://www.cuilab.cn/hmdd, accessed date on 15 October 2021) database, which [27] is a manual collection of human miRNA-disease association database. Human miRNA-disease related information has been experimentally confirmed. The detailed data are indicated in Table 1.

We construct the adjacency matrix $Y\in \;R^{p\times q}$, which is composed of disease $d_{i}\left(1\leq i\leq p\right)$ and miRNA $m_{j}\left(1\leq j\leq q\right)$, the matrix $Y\in \;R^{p\times q}$ is defined as Equation (1):

(1) $$Y\left(d_{i},m_{j}\right)=\{\begin{array}{c} 1\;\;\;\;\;\;\;\;\;\;\;\;\;\;\;\;Disease\;d_{i}\;is\;related\;to\;\;miRNA\;m_{j}\;\;\;\\ -1\;\;\;\;\;\;\;\;\;\;\;\;\;\;Disease\;d_{i}\;is\;not\;related\;to\;\;miRNA\;m_{j} \end{array}$$

### 2.2. MiRNA Functional Similarity

There are interactions between miRNAs, which will affect various biological processes. Wang et al. [28] use the MISIM method to determine the functional similarity scores of miRNAs. We construct a miRNA functional similarity adjacency matrix with 495 rows and 495 columns. Each element in the matrix indicates the functional similarity score between two miRNAs.

### 2.3. Disease Semantic Similarity

The U.S. National Library of Medicine’s MeSH (http://www.ncbi.nlm.nih.gov/, accessed date on 15 October 2021) provides the disease semantic similarity information. MeSH [29] has so far collected more than 18,000 medical keywords, which are divided into 16 categories. Among them, category C has a strict classification of diseases, which is more conducive for future research on the diseases. Each disease is represented by a directed acyclic graph (DAG), where the dots in the DAG represent a disease, and the edges represent the relationship between diseases.

According to the hypothesis, the similarity of the two disease is associated with the shared items, so based on the DAG of diseases, Wang et al. [28] proposed a method to calculate the semantic similarity of diseases, which is defined as follows:

(2) $$D_{d\left(i\right)}\left(t\right)=\{\begin{array}{ll} 1 & if\;t=d\left(i\right)\\ \;max\left\{\Delta *D_{d\left(i\right)}\left(t^{\prime }\right)|t^{\prime }\;\epsilon \;chidren\;oft\right\} & if\;t\neq d\left(i\right) \end{array}$$

(3) $$DV\left(d\left(i\right)\right)=\sum_{t\epsilon T_{d\left(i\right)}}D_{d\left(i\right)}\left(t\right)$$

Equation (2) demonstrates the semantic score of disease t, $T_{d\left(j\right)}$ is the node set, $E_{d\left(i\right)}$ is the corresponding link set.

We usually use ∆ to denote the semantic contribution factor, and specific scores are calculated using Equation (3) for the semantic score of disease d(i), where the contribution of disease d(i) to its own semantic value is 1, and other ancestral diseases gradually decrease their contribution to the semantic value of disease d(i) as their distance from disease d(i) increases.

Therefore, the semantic similarity between the two diseases d(i) and d(j) can be calculated by Equation (4):

(4) $$K_{d,sem}\left(d\left(i\right),d\left(j\right)\right)\;=\frac{\sum_{t\epsilon T_{d\left(i\right)}\cap^{}T_{d\left(j\right)}}(D_{d\left(i\right)}\left(t\right)+D_{d\left(j\right)}\left(t\right))}{DV\left(d\left(i\right)\right)+DV\left(d\left(j\right)\right)}$$

View reference Ding et al. [23], where we can see more details about above equation.

### 2.4. Method Models

#### 2.4.1. Collaborative Matrix Factorization

The first sub-model used in the experiments is Collaborative matrix factorization (CMF) model [24] which is a classic baseline, it is often used for comparison in recommendation system related studies such as rating prediction and cold-start recommendations. The formulas of the CMF model are as follows:

(5) $$Y\approx \;AB^{T}$$

Then minimize the squared error of our objective function:

(6) $$arg\underset{A,B}{min}{∥Y-AB^{T}∥}_{F}^{2}\;,$$

(7) $$\;\;S_{m}\approx \;AA^{T},\;S_{d}\approx \;BB^{T}$$

where $∥.{∥}_{F}$ is Frobenius norm, matrix A is the matrix of miRNAs features and matrix B is the matrix of diseases features. Finally, the matrix of predicted miRNA-disease interactions F is calculated by Equation (8).

(8) $$F=AB^{T}$$

#### 2.4.2. Neighborhood Regularized Logistic Matrix Factorization

The second sub-model is Neighborhood Regularized Logistic Matrix Factorization (NRLMF) [25], which is a common approach in machine learning. It predicts associations by combining logistic matrix factorization (LMF) and domain regularization. Some of the equations that will be used in the model are as follows:

(9) $$\underset{U,V}{min}\overset{m}{\underset{i=1}{\sum }}\overset{n}{\underset{j=1}{\sum }}\left(1+cy_{ij}-y_{ij}\right)ln\left[1+exp\left(u_{i}v_{j}^{T}\right)\right]-cy_{ij}u_{i}v_{j}^{T\;}+\frac{1}{2}tr\left[U^{T}\left(\lambda_{d}I+\alpha L^{d}\right)U\right]+\frac{1}{2}tr\left[V^{T}\left(\lambda_{t}I+\beta L^{t}\right)V\right]$$

where $P\in R^{m\times n}$, In the algorithm, the objective function of Equation (9) is denoted by L, and the partial gradients with respect to U and V are listed in the following equation:

(10) $$\frac{\partial L}{\partial U}=PV+\left(c-1\right)\left(Y⊙P\right)V-cYV+\left(\lambda_{d}I+\alpha L^{d}\right)U$$

(11) $$\frac{\partial L}{\partial V}=P^{T}U+\left(c-1\right)\left(Y^{T}⊙P^{T}\right)U-cY^{T}U+\left(\lambda_{t}I+\beta L^{t}\right)V$$

where the (i,j) element is $P_{ij}$, ⊙ denotes the Hadamard product of two matrices.

#### 2.4.3. Laplacian Regularized Least Squares

The third sub-model used in the experiments is Laplacian Regularized Least Squares (LapRLS) [26], which is a common prediction model in machine learning and it belongs to semi-supervised learning methods. We build the flow model via building a nearest neighborhood graph. Then by introducing the Laplacian graph in the least square loss function coefficients to achieve the regularization purpose. Some of the equations that will be used are as follows:

(12) $$F_{d}^{*}=\underset{F_{d}}{min}J(F_{d})={∥Y-F_{d}∥}_{F}^{2}+\beta_{d}Trace\left(F_{d}^{T}L_{d}F_{d}\right)$$

(13) $$F_{d}^{*}=W_{d}\alpha_{d}^{*}\;$$

The next step is to tell the derivative of the objective function, which will vanish at the minimization.

(14) $$-W_{d}\left(Y-W_{d}\alpha_{d}\right)+\beta_{d}\alpha_{d}^{T}W_{d}L_{d}W_{d}\alpha_{d}=0\;$$

Then we can obtain the following equation:

(15) $$\alpha_{d}^{*}={\left(W_{d}+\beta_{d}L_{d}W_{d}\right)}^{-1}Y$$

In the end, we can get:

(16) $$F_{d}^{*}=W_{d}{\left(W_{d}+\beta_{d}L_{d}W_{d}\right)}^{-1}Y$$

(17) $$F_{m}^{*}=W_{m}{\left(W_{m}+\beta_{m}L_{m}W_{m}\right)}^{-1}Y^{T}$$

(18) $$F^{*}=\frac{F_{d}^{*}+F_{m}^{*}{}^{T}}{2}$$

$W_{d}$ is the weight of the disease and $W_{m}$ is the weight of the miRNA, This helps us to calculate the results later.

It is used because it is simple and its performance is comparable to that of Laplacian regularized support vector machines. LapRLS depends on the regularization term of the data being a normalized Laplacian operation on the graphs.

#### 2.4.4. Reinforcement Learning

Nowadays, machine learning has become a common computational method in research, and reinforcement learning plays an essential role in machine learning. In reinforcement learning, we use four main elements: agent, reward, environment state and action. Agent manipulates the environment by taking action and moving from this moment state to the next state. If the task is finished, the agent is given a positive reward. If not, it is given a negative reward. The purpose of reinforcement learning is to gain cumulative more rewards.

In reinforcement learning, Q-learning algorithm is a commonly used algorithm that is value-based, where the Markov problem will be solved with Bellman′s equation and off-policy learning by using the time difference method. Q is Q(s,a), that is, in a certain state, doing an action a, an immediate reward r will be given back, and the environment will also give the corresponding rewards depending on the agent’s action. Therefore, the algorithm stores Q-values by constructing a Q-table of states and actions, then chooses the action that will yield the maximum benefit upon the Q-values.

#### 2.4.5. RFLMDA

We perform association prediction based on reinforcement learning, we divide the dataset into training set, validation set and test set in the ratio of 8:1:1 and validate the performance with five-fold cross-validation. First, three sub-models are used, namely CMF [24], NRLMF [25] and LapRLS [26], which are trained on the training set, and then the three sub-models are fused with models via the Q-learning algorithm. In the Q-learning section, the weights occupied by the three sub-models themselves are set as the state space S, and the weight change values of the weights occupied by the three sub-models are set as the action space A. $F^{*}$ is iteratively updated on the verification set, and each round generates a new AUC. We use the difference of AUC as the reward benchmark, if the value of the difference between the next state′s and this state′s AUC is larger than 0, we will give a reward of plus 1. Otherwise, give a reward of minus 1. With continuous iterative training, the Q values converge continuously and the three parameters approach the optimal solution. Finally tested in the test set, we obtained the weight value S  (0.1735, 0.2913, 0.5352) and the AUC’s value (0.9416). Therefore, the RFLMDA model gets better results.

**Figure 1:**
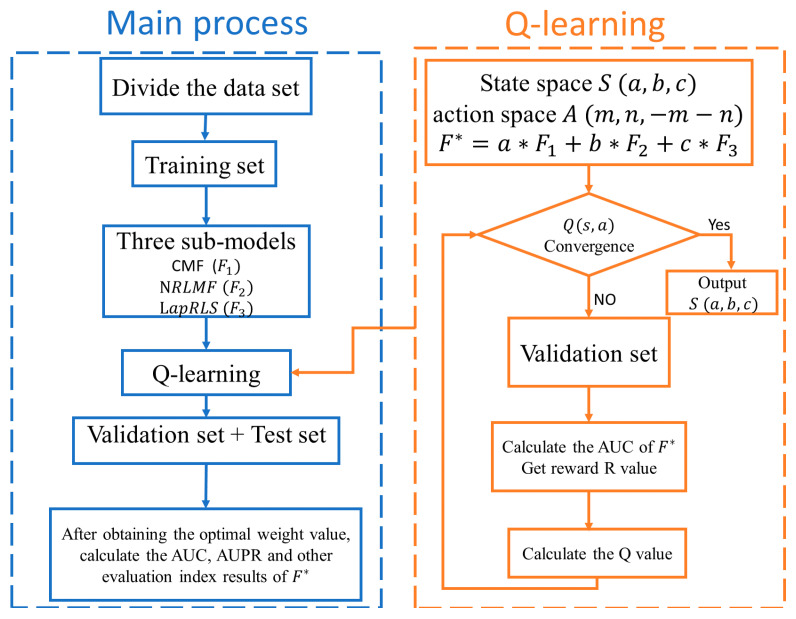
Overall flow chart of RFLMDA.

Pseudocode for RFLMDA algorithm is list in Algorithm 1. The pseudocode for Q-learning is listed in Algorithm 2. Overall flow chart of RFLMDA is shown in Figure 1.
**Algorithm1:** Pseudocode for RFLMDA algorithm.**Require:** Action space A, state space S, reward value R, sub-models CMF, NRLMF and LapRLS.
**Ensure:** The predicted results of
$F^{*};$
1: Processing the dataset and training sub-models, namely CMF, NRLMF and LapRLS, respectively;
2: Calculation of the weights for models $F_{1},F_{2}\;and\;F_{3}$ via Pseudocode for Q-learning algorithm, respectively;
3: Combining $F_{1},\;F_{2},\;F_{3}$ and S(a, b, c)$\;\mathrm{by}\;\;F^{*}=a*F_{1}+b*F_{2}+c*F_{3}$.
**Algorithm2:** Pseudocode for Q-learning algorithm.
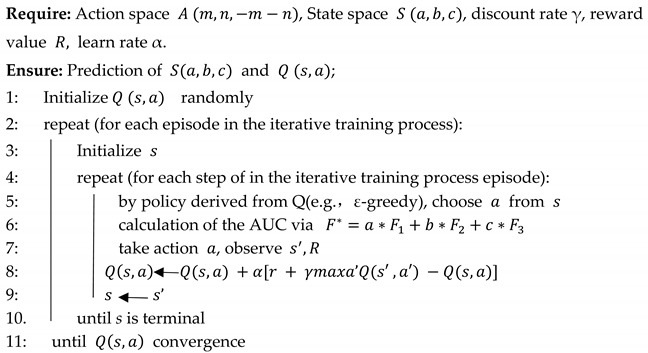


## 3. Results

### 3.1. Evaluation Measurements

The area under the PR curve is called AUPR (area under the PR curve). The PR curve (precision recall curve) is a curve derived from the concepts of Precision check accuracy rate and Recall check completeness, with Recall on the *X*-axis and Precision on the *Y*-axis.

AUC (Area Under Curve) is the area of the plane graph enclosed by the ROC curve and the abscissa axis, and its value is between 0 and 1. When it is equal to 0.5, the evaluation is the lowest and there is no use value. As it gets closer to 1, the better the model is. In practical applications, the performance advantages and disadvantages of different statistical models can be compared by comparing the AUC values of the ROC curves of different statistical classification models.

**Figure 2:**
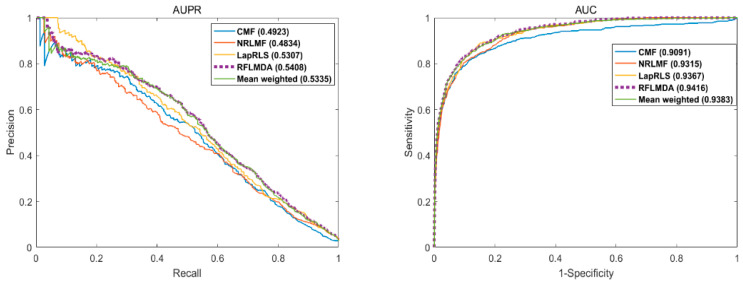
AUPR and AUC of RLFMDA and other methods in five-fold cross-validation.

We use AUC and AUPR as evaluation measurements, and compare the performance of RFLMDA with Mean weighted, CMF [24], NRLMF [25], and LapRLS [26]. The Mean Weighted method is to assign 1/3 of the weight value to all three submodels, Mean Weighted in order to compare with reinforcement learning methods. Observe the change of experimental results of three submodels under the same weight. In this way, we verify the necessity of applying reinforcement learning algorithm. By the five-fold cross-validation, RLFMDA, Mean Weighted, CMF, NRLMF and LapRLS obtained AUC values of 0.9416, 0.9383, 0.9091, 0.9315, 0.9367 respectively. Figure 2 is the obtained result graph. 

**Figure 3:**
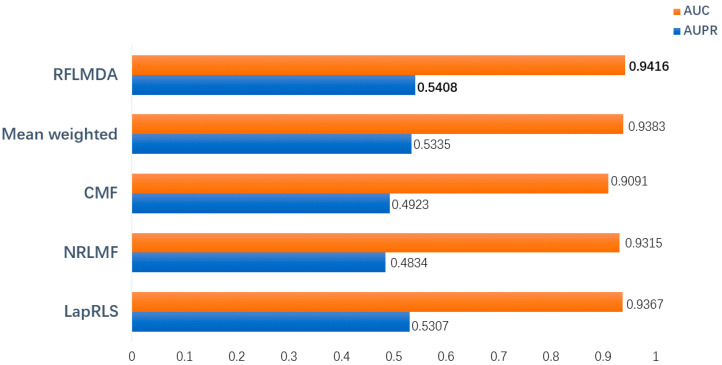
Comparison of RFLMDA with other methods.

Figure 3 shows the bar chart of AUC for the five methods. From the results, it is clear that the RFLMDA model has better predictive performance.

### 3.2. Comparison with Other Methods

**Figure 4:**
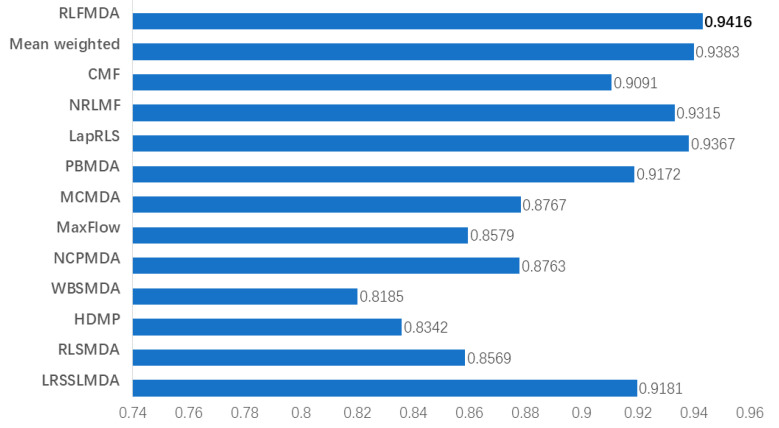
AUC of 13 methods via five-fold cross-validation.

To further validate the model performance, we conducted a comparison experiment. We compare the RFLMDA model with other 12 methods, including CMF [24], NRLMF [25], LapRLS [25], PBMDA [30], MCMDA [31], MaxFlow, NCPMDA [32], WBSMDA [19], HDMP [15], RLSMDA [18], LRSSLMDA [33], Mean weighted. The comparative results are shown in Figure 4. The weight obtained by the experiment is S (0.1735, 0.2913, 0.5352). The AUC of RFLMDA is 0.9416, which is better than other methods. It can be seen that RLFMDA has the best effect.

## 4. Case Study

In this section, we perform a case study, to further evaluate the model RFLMDA prediction performance. The case study method can objectively and effectively evaluate the predictive performance of statistical models in a more in-depth manner.

Therefore, we select 8 common diseases for local verification to predict unknown miRNA-disease associations in HMDD via known miRNA-disease associations contained in HMDD. Two independent databases (i.e., dbDEMC [34] and miR2Disease [35]) were used as benchmarks. The prediction results are verified by this dataset. The verification results of the top 50 lists are summarized in Table 2.

All the top 50 miRNAs associated with Colorectal Neoplasms and Breast Neoplasms have been confirmed. We used every known miRNA-disease association as a test sample, and the training samples were other known miRNA-disease associations. In the absence of any evidence of a known association, the test samples were classified as candidate miRNA-disease associations. Among the top 50 miRNAs related to Gastric Neoplasms, Colon Neoplasms, Pancreatic Neoplasms, Esophageal Neoplasms, Kidney Neoplasms and Lymphoma, we confirm 41, 47, 49, 46, 46, and 48 miRNAs respectively.

Next, we also conduct a detailed analysis of Colorectal Neoplasms, Breast Neoplasms and Lymphoma.

### 4.1. Colorectal Neoplasms

Colorectal Neoplasms is common malignant tumors. Because of abnormal production of cells, it may attack or spread to other body parts. Most of them develop in the lining of the intestine and rectum, usually starting as polyps. These polyps are benign growths and most are harmless, but if they remain undetected, they may become cancerous. In Singapore, colorectal Neoplasms is the most prevalent cancer in men, and the most prevalent in people over 50 years of age.

The validation results are in Table 3. From the confirmed results, we can see that among the top 20 miRNAs related to colorectal Neoplasms, all of them have been confirmed in the dbDEMC or HMDD dataset.

### 4.2. Breast Neoplasms

Breast Neoplasms is a tumor that occurs in breast tissue, and accounts for about 2/3 of breast diseases. Malignant breast neoplasms are usually called breast cancer, 99% of which occur in women, which is now a common disease that endangers the health of women worldwide. It is predicted that most women are diagnosed in the advanced stage of breast cancer. Therefore, in order to treat the disease in the early stage, it is urgent to further decipher the pathogenesis of breast neoplasms.

In previous studies, it can be known that miRNAs are closely associated with Breast Neoplasms. For example, the let-7 family was mainly a Neoplasms suppressor that inhibits the development and migration of breast cancer. In the evaluation of breast Neoplasms, the top 20 alternate miRNAs were potentially related to breast Neoplasms were selected, all of which are confirmed by the dataset. The validation results are in Table 4.

### 4.3. Lymphoma

Lymphoma is the most prevalent type of blood cancer and it originates from the lymphopoietic system, and it usually refers to the rapid and uncontrolled growth of abnormal lymphocytes. Lymph is an immune organ that spreads all over the body. Once it becomes cancerous, the impact on human life and health is quite serious. Around the world, about 1000 people are diagnosed with lymphoma every day.

We performed local validation for lymphoma and obtained the predicted results shown in Table 5. In the top 20 predicted miRNAs, all of them have been confirmed in the dbDEMC or HMDD dataset.

In conclusion, it shows that our method plays a role in predicting association information between miRNAs and human diseases, and which is a trustworthy model for association prediction.

## 5. Conclusions and Discussion

In this work, we combine Q-learning algorithm of reinforcement learning to propose a RFLMDA model, fusing three submodels CMF [24], NRLMF [25] and LapRLS [26] are fused via Q-learning algorithm. Then multiple rounds of iterative updates are performed to obtain the optimal weight S. The performance of RFLMDA was evaluated via five-fold cross-validation and local validation. As a result, the optimal weight is obtained as S (0.1735,0.2913,0.5352), and the AUC is 0.9416. By comparing the experiments with other methods, it is proved that RFLMDA model has better performance.

In order to further validate the predictive performance of RFLMDA, we use eight diseases for local verification and conducted case study on three common human diseases. As a result, all the top 50 miRNAs related to Colorectal Neoplasms and Breast Neoplasms have been confirmed. Among the top 50 miRNAs related to Gastric Neoplasms, Colon Neoplasms, Pancreatic Neoplasms, Esophageal Neoplasms, Kidney Neoplasms, and Lymphoma, we confirm 41, 47, 49, 46, 46, and 48 miRNAs respectively.

The above results suggest that our proposed RFLMDA is a reliable model and can provide high-confidence miRNA candidates for biological experiments. In our future work, we hope that further improvements will be made to the existing algorithm and expect better prediction results.

In comparison to existing technology, our methodological improvement is to optimize the performance and program running speed of miRNA-disease association prediction. The potential benefit is to provide a new direction for future miRNA-disease association prediction accuracy, which could advance the development of human disease therapy and gene pharmaceuticals. In the future, we will consider other optimization algorithms in reinforcement learning to build related models to see if we can further improve the performance of miRNA-disease association prediction.

## Figures and Tables

**Table 1:** Statistics of associated information.

Type of Data	Quantity
MiRNAs	495
Diseases	383
MiRNA-Disease association	5430

**Table 2:** The Top-50 prediction list of 8 common human diseases.

Disease Name	Top-50 Prediction List
Colon Neoplasms	47
Kidney Neoplasms	46
Pancreatic Neoplasms	49
Esophageal Neoplasms	46
Breast Neoplasms	50
Gastric Neoplasms	41
Lymphoma	48
Colorectal Neoplasms	50

**Table 3:** Top 20 miRNAs predicted by the RLFMDA model to be associated with Colorectal Neoplasms.

Disease	Rank	Name	Evidence	Rank	Name	Evidence
Colorectal Neoplasms	1	mir-21	D	11	mir-7	D
	2	mir-145	D	12	mir-218	D
	3	mir-210	D	13	mir-148a	D
	4	mir-182	D	14	mir-27a	H
	5	mir-196a	D	15	mir-133a	D
	6	mir-126	D	16	mir-143	D
	7	mir-30a	D	17	mir-31	D
	8	mir-34a	D	18	mir-200c	D
	9	mir-183	D	19	mir-34b	D
	10	mir-146b	H	20	mir-7	D

In the table, HMDD is represented by H and dbDEMC is represented by D.

**Table 4:** Top 20 miRNAs predicted by the RLFMDA model to be associated with Breast Neoplasms.

Disease	Rank	Name	Evidence	Rank	Name	Evidence
Breast Neoplasms	1	let-7f	D	11	mir-10b	D
	2	mir-30c	D	12	mir-19a	D
	3	mir-22	D	13	mir-302b	D
	4	mir-17	D	14	mir-200c	D
	5	mir-34c	H	15	let-7g	D
	6	mir-18a	D	16	mir-29a	D
	7	let-7a	D	17	mir-191	D
	8	mir-20a	D	18	mir-125a	D
	9	mir-218	D	19	mir-151a	H
	10	mir-34b	H	20	mir-200b	D

In the table, HMDD is represented by H and dbDEMC is represented by D.

**Table 5:** Top 20 miRNAs predicted by the RLFMDA model to be associated with lymphoma.

Disease	Rank	Name	Evidence	Rank	Name	Evidence
Lymphoma	1	mir-17	D	11	mir-146a	D
	2	mir-20a	D	12	mir-34a	D
	3	mir-19b	D	13	mir-125b	D
	4	mir-92a	D	14	mir-126	D
	5	mir-18a	D	15	mir-145	D
	6	mir-21	D	16	mir-181a	D
	7	mir-19a	D	17	mir-24	D
	8	mir-155	D	18	mir-29b	D
	9	mir-16	D	19	mir-101	D
	10	mir-15a	D	20	mir-150	D

In the table, HMDD is represented by H and dbDEMC is represented by D.

## Data Availability

Data and code can be requested from the corresponding author.
